# Injectable Glycol Chitosan Hydrogel Containing Folic Acid-Functionalized Cyclodextrin-Paclitaxel Complex for Breast Cancer Therapy

**DOI:** 10.3390/nano11020317

**Published:** 2021-01-27

**Authors:** Hoon Hyun, Min Ho Park, Gayoung Jo, Bo Young Lee, Jae Won Choi, Heung Jae Chun, Hyeon Soo Kim, Dae Hyeok Yang

**Affiliations:** 1 Department of Biomedical Sciences, Chonnam National University Medical School, Gwangju 61469, Korea; hhyun@jnu.ac.kr (H.H.); 196729@jnu.ac.kr (G.J.); mhpark@jnu.ac.kr (B.Y.L.); 2 Department of Surgery, Chonnam National University Medical School, Gwangju 61469, Korea; 176250@jnu.ac.kr; 3 Lumenbio Co., LTD., Seoul 08590, Korea; aa@lumenbio.co.kr; 4 Department of Biomedical & Health Sciences, College of Medicine, The Catholic University of Korea, Seoul 06591, Korea; chunhj@catholic.ac.kr; 5 Institute of Cell and Tissue Engineering, College of Medicine, The Catholic University of Korea, Seoul 06591, Korea; 21905010@cmcnu.or.kr

**Keywords:** glycol chitosan, beta-cyclodextrin, polyethylene glycol, folic acid, breast cancer therapy

## Abstract

We prepared a drug carrier which consisted of injectable methacrylated glycol chitosan (MGC) hydrogel, and a conjugate of 6-monodeoxy-6-monoamino-β-cyclodextrin⋅hydrochloride (6-NH_2_-β-CD⋅HCl), polyethylene glycol (PEG), and folic acid (FA) for the local delivery and improved cellular uptake of paclitaxel (PTX) (MGC/CDPF-ic-PTX). CDPF refers to a conjugate of 6-NH_2_-β-CD⋅HCl, PEG, and FA. The anti-cancer effect was investigated using a xenograft mouse model. As controls, the animal study on MGC/PTX and MGC/CD-ic-PTX was performed. The swelling ratio of all samples was analyzed for 7 days, and it showed a gradual increase for 3 days and a maintained state afterward. From the release result, the MGC-based samples have an initial burst for 1 day and a sustained release for 7 days. Results of cytotoxicity and animal study showed the biocompatibility and superior anti-cancer effect of MGC/CDPF-ic-PTX against breast cancer. Furthermore, histological results showed the anti-cancer capacity of MGC/CDPF-ic-PTX against breast cancer. These findings suggest that MGC/CDPF-ic-PTX has clinical potential for breast cancer therapy.

## 1. Introduction

Local drug delivery systems, which are intratumorally implanted and adjacent to cancer tissues, are suitable for effective cancer treatment because they can achieve high therapeutic capacity resulting from the high concentration of anti-cancer drugs around cancer tissues, followed by minimizing normal tissue toxicity and improving low bioavailability [1,2,3,4,5]. Therefore, to date, several kinds of platforms, such as films for releasing drugs, gels, wafers, rods, and particles, have been designed for localized drug delivery systems [1].

Among these platforms, hydrogels have attracted much attention because of their injectability, along with ease of drug targeting to cancer tissue and minimal toxicity to normal tissues [1,2,3,4,5,6,7]. Furthermore, these injectable hydrogels improve the water solubility of anti-cancer drugs, thereby decreasing the required number of drugs and increasing the amount that reaches the targeted sites [1,2,3,4,5,6,7]. These characteristics are closely related to the improvement in targeted drug delivery.

Nevertheless, poor targeting of cancer cells by many anti-cancer drugs can lead to low therapeutic efficacy. Therefore, new approaches, such as conjugation of ligands to the drugs and encapsulation of the drugs into ligand-decorated nano-based materials, have been attempted because the ligands can improve binding of the cancer cell membranes and induce high intracellular uptake of the drugs by receptor-mediated endocytosis, leading to an improvement in the target cancer therapy [8,9,10,11].

Here, we fabricated a local system for paclitaxel (PTX) delivery against breast cancer, which was made of injectable methacrylated glycol chitosan (MGC) hydrogel photopolymerizable by visible light irradiation and folic acid/polyethylene glycol (PEG)-conjugated beta-cyclodextrin (CDPF), which was designated as MGC/CD-ic-PTX. The MGC hydrogel and the CDPF were used as a reservoir for containing PTX and specifically targeting the drug to cancer cells. PTX is a drug that is clinically used for breast cancer therapy [12]. However, the poor water-solubility of PTX leads to low bioavailability; therefore, beta-cyclodextrin (β-CD) was used for improving the water solubility of PTX by inclusion complex formation [13].

Conjugation of a methacrylic group to the GC backbone leads to a photopolymerizable hydrogel using visible-light irradiation. For CDPF, folic acid (FA) and β-CD were used as a ligand for cell targeting and reservoir for drug loading, respectively [10,11,14,15,16]. The anti-cancer efficacy of MGC/CDPF-ic-PTX using a tumor-bearing xenograft mouse model was evaluated.

## 2. Materials and Methods

### 2.1. Materials

Glycol chitosan (GC; ≥60% from titration) with crystallinity and 585,000 g/mol and purchased from glycidyl methacrylate (GM), 6-monodeoxy-6-monoamino-beta-cyclodextrin⋅hydrochloride (6-NH_2_-β-CD⋅HCl), beta-cyclodextrin (β-CD), triethylamine (TEA), and coumarin 6 (Cou6) were purchased from Sigma–Aldrich (St. Louis, MO, USA). Riboflavin 5′-monophosphate sodium salt (riboflavin) as a visible light photo initiator was supplied by Santa Cruz Biotechnology, Inc. (Santa Cruz, CA, USA). Paclitaxel, used for breast cancer therapy, was obtained from Shin Poong Pharm, Co., Ltd. (Ansan, Kyunggi, Republic of Korea). Folic acid polyethyleneglycol acid (FA-PEG-COOH, Mw: 3400 g/mol) was supplied by Nanocs Inc. (New York, NY, USA). HOOC-PEG-OH (Mw: 3400 g/mol) was supplied by Creative PEGWorks (Chapel Hill, NC, USA). 4-(4,6-Dimethoxy-1,3,5-triazin-2-yl)-4-methylmorpholinium chloride (DMT-MM; Osaka, Japan) was used as a condensing agent for conjugation of 6-NH_2_-β-CD⋅HCl to FA-PEG-COOH. Cellulose membranes (cut-off: 2.5 kDa) were purchased from Spectrum Laboratories Inc. (Rancho Dominguez, CA, USA). The NIH3T3-E1 mouse fibroblast and MCF-7 human breast cancer cell lines were obtained from American Type Culture Collection (ATCC; Manassas, VA, USA). Cell counting kit-8 (CCK-8; Dojindo Molecular Technologies, Inc. Rockville, MD, USA) was used for the in vitro cell viability assay. Received chemicals were used without further purification.

### 2.2. Inclusion Complex Formation between 6-NH_2_-β-CD⋅HCl-Conjugated FA-PEG/β-CD and PTX (CDPF-ic-PTX and CD-ic-PTX)

#### 2.2.1. Preparation of CDPF and 6-NH_2_-β-CD⋅HCl-conjugated PEG (CDP)

FA-PEG-COOH (0.3 mmol, 1 g) and DMT-MM (0.5 mmol, 138 mg) were dissolved in DMSO (20 mL) and stirred for 1 h. After adding 6-NH_2_-β-CD⋅HCl (0.5 mmol, 585 mg) and TEA (0.5 mmol, 51 mg), the mixture was reacted at room temperature for 24 h. To remove unreacted 6-NH_2_-β-CD⋅HCl, DMT-MM, salt, and TEA, the mixture was dialyzed (cut-off: 2.5 kDa). The reactant was lyophilized at −90 °C for 7 days, followed by storing in a refrigerator (−20 °C) (Yield: 85.5%). CDP was prepared according to the preparation method of CDPF. For CDP preparation, HOOC-PEG-OH (0.3 mmol, 1 g), DMT-MM (0.5 mmol, 138 g), and 6-NH_2_-β-CD⋅HCl (0.5 mmol, 51 mg) were used (Yield: 88.2%).

#### 2.2.2. CDPF-ic-PTX, CD-ic-PTX, and CDP-ic-PTX

CDPF-ic-PTX was formed by dropping PTX solution (0.012 mmol, 10 mg) dissolved in acetone (1 mL) into CDPF solution (0.012 mmol, 41 mg) in a co-solvent of DMSO and water (10 mL, 50:50 *v/v*%). After dropping, the mixture was stirred for 48 h to evaporate the organic solvent and then dialyzed using a dialysis membrane tube (1 kDa) to eliminate DMSO. To eliminate uncomplexed PTX, the mixture was centrifuged and filtered using a filter paper. The mixture was then lyophilized and stored in a desiccator (Yield: 90.1%). CD-ic-PTX and CDP-ic-PTX were formed according to the CDPF-ic-PTX preparation protocol. In common with CDPF and PTX of CDPF-in-PTX, the same moles of CD, CDP, and PTX were used (Yield: 93.5% and 91.3%). The complexed PTX amount of CDPF-ic-PTX, CD-ic-PTX, and CDP-ic-PTX in DMSO was calculated using Ultraviolet-visible (UV-vis) spectroscopy (Multiskan^®^ Spectrum; Thermo Fisher Scientific; Waltham, MA, USA) at 227 nm.

#### 2.2.3. Analyses

Conjugation of 6-NH_2_-β-CD⋅HCl to FA-PEG-COOH and inclusion complexes between CDPF/CD and PTX were analyzed using proton nuclear magnetic resonance (^1^H NMR; Bruker Avance; Bruker Avance 400; Harwell, UK) with D_2_O. The inclusion complexes were analyzed using UV-vis spectroscopy (Multiskan^®^ Spectrum; Thermo Fisher Scientific; Waltham, MA, USA) using PBS (pH 7.4), which were measured in quartz cuvettes (frosted wall, 0.7 mL). Their thermal behaviors were analyzed using a differential scanning calorimeter (DSC; TA Instruments; DSC Q2000; New Castle, DE, USA). The samples (50 mg), heated in an aluminum pan, were monitored from 50 °C to 250 °C at 10 °C/min.

### 2.3. Preparation of Methacrylated GC (MGC)

MGC was prepared according to our previous studies [2,3,4,5,17]. Glycidyl methacrylate (GM; 0.05 mmol, 7 mg) was added to an aqueous GC solution (0.003 mmol, 1.5 g; 500 mL), followed by adjustment to pH 9. After reacting at room temperature for 2 days, the mixture was neutralized. After dialysis (cut-off: 20 kDa) for 7 days in water, the purified solution was filtered and lyophilized at −90 °C for an additional 7 days before use.

### 2.4. Preparation of Injectable PTX-Loaded and CD/CDPF-ic-PTX-Loaded MGC Hydrogel (MGC/PTX, MGC/CD-ic-PTX, and MGC/CDPF-ic-PTX)

CDPF-ic-PTX (PTX–2 mg/mL) was gently dropped into a solution of MGC (1 *w*/*v*%) and riboflavin (12 μM). The mixture was irradiated for 10 s using a blue light (430–485 nm, 2100 mW/cm^2^, light-emitting diode curing light, Foshan Keyuan Medical Equipment Co., Ltd., Foshan, China) for gelation. MGC/PTX and MGC/CD-ic-PTX were also prepared in the same way as MGC/CDPF/PTX.

### 2.5. Swelling Ratios of MGC/PTX, MGC/CD-ic-PTX, and MGC/CDPF-ic-PTX

The swelling ratio and degradation ratio were calculated as follows:

Swelling ratio = The swollen weight at each time interval/The initial weight of each hydrogel

Degradation ratio = The weight of hydrogels at time intervals/The initial weights of hydrogel

### 2.6. In Vitro Release Test of PTX in MGC/PTX, MGC/CD-ic-PTX, and MGC/CDPF-ic-PTX

Cellulose membrane tubes (cut-off: 3500 g/mol) containing MGC/PTX (PTX −2 mg/mL), MGC/CD-ic-PTX (PTX −2 mg/mL), and MGC/CDPF-ic-PTX (PTX: 2 mg/mL) were added in conical tubes and dialyzed using phosphate buffered saline (PBS; pH 7.4, 8 mL). This setting was incubated at 37 °C with continuous agitation at 100 rpm. At predetermined time intervals (1, 2, 3, 5, 7, 12, 18, 24, 48, 72, 96, 120, 144, and 168 h), 2 mL of efflux was extracted, and the sample volume of PBS was supplemented. High-performance liquid chromatography (HPLC) was employed for investigating the released amount of PTX. Test conditions were as follows; mobile phase: acetonitrile and water (50:50, *v/v*%); injection volume: 20 μL; UV detector (1100 series, Agilent Technologies, Palo Alto, CA, USA), and an Ascentis C18 column (25 cm × 4.6 mm, particle size: 5 µm; Supelco, St. Louis, MO, USA). PTX was detected at 227 nm.

### 2.7. In Vitro Cell Proliferation and Flow Cytometry Assays

The MCF-7 cell line was cultured with Dulbecco’s Modified Eagle Medium (DMEM) containing fetal bovine serum (FBS; 10%) and penicillin/streptomycin (10 mg/mL; 1%). The cells were passaged in the case of 80% confluence. For this test, the cells between 7 and 12 passages were used. The media was changed every three days. The cell proliferation rates of CDPF ranged from 0 to 500 μg/mL and five samples, including control, PTX, MGC/PTX, MGC/CD-ic-PTX, and MGC/CDPF-ic-PTX, were evaluated. In the case of PTX, MCF-7 cells (5 × 10^3^ cells/well) were added to each well and incubated at 37 °C for 3 h to adhere to the cells. Afterward, the cell-adhered well surface was treated with PTX (2 mg/mL) and then incubated for 1, 3, 5, and 7 days. The medium containing PTX (2 mg/mL) was changed every three days to maintain a constant PTX concentration. In the case of the hydrogel-based samples, MGC/PTX, MGC/CD-ic-PTX, and MGC/CDPF-ic-PTX hydrogels were first formed on each well. After seeding the sample amounts of the cells, they were incubated at 37 °C for 1, 3, 5, and 7 days. The media was changed every three days. At each time point, PBS (pH 7.4) washed cells were treated with CCK-8 (10 μL) and afterward incubated for an additional 4 h. The cells’ absorbance was measured at 450 nm. For flow cytometry analysis, Cou6 was used in place of PTX because coumarin 6 has fluorescence and forms inclusion complex formation with β-CD derivatives [18,19]. The cellular uptakes of CDPF-ic-Cou6, CDP-ic-Cou6, and CD-ic-Cou6 against MCF-7 cells were evaluated by flow cytometry analysis. In addition, the cellular uptake of CDPF-ic-Cou6 against NIH3T3-E1 cells was also investigated. The two types of cells (1 × 10^5^ cells/mL) were treated to the used samples for 4 h and then washed with PBS (pH 7.4) three times. Afterward, the cells were harvested with 0.25% trypsin/ethylenediaminetetraacetic acid (EDTA) and then transferred to 5 mL tubes. A total of 10,000 cells were acquired for this analysis.

### 2.8. In Vivo Animal Test

**Figure 1:**
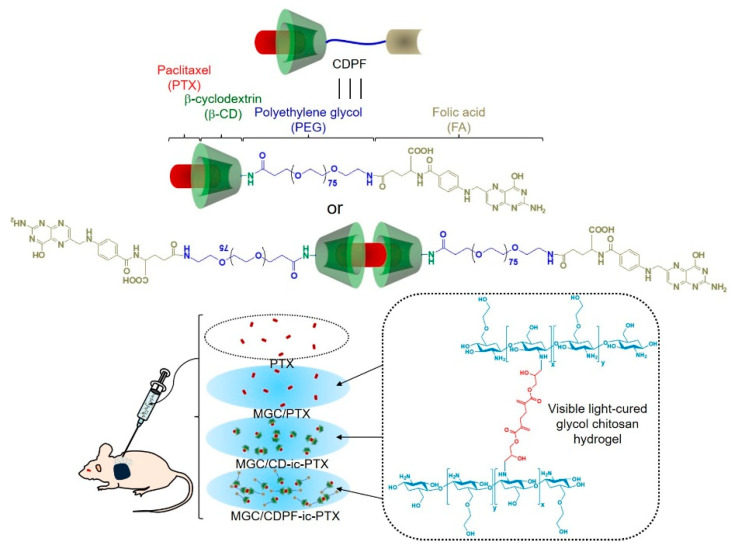
Schematic illustration showing in vivo animal test. Total four samples, paclitaxel (PTX), methacrylated glycol chitosan MGC/PTX, MGC/CD-ic-PTX, and MGC/CDPF-ic-PTX, were locally injected near tumor tissue. As a comparative study, no treatment was used as a control. CD—6-monodeoxy-6-monoamino-β-cyclodextrin⋅hydrochloride (6-NH_2_-β-CD⋅HCl); CDPF—a conjugate of 6-NH_2_-β-CD⋅HCl, polyethylene glycol (PEG), and folic acid (FA).

This animal test was approved by the Chonnam National University Animal Research Committee (CNU IACUC-H-2017-64). Figure 1 illustrates the animal test using PTX, MGC/PTX, MGC/CD-ic-PTX, and MGC/CDPF-ic-PTX. For this test, MCRNU nude mice (22–25 g; Seong nam, Republic of Korea), 6 weeks old, were used (*n* = 6 per group and time point). MCF-7 cells (5 × 10^6^ cells) were suspended in water for injection (100 µL; Jeil Pharmaceutical Co. Ltd., Daegu, Korea) and transferred to a 1-mL insulin syringe. After injecting the suspension into the back of each mouse, the animals were cared for until the tumor reached approximately 1 cm. PTX (2 mg/kg) was systemically injected via the tail vein of the mice. Because of its poor water solubility, PTX (2 mg/kg) was dissolved in the co-solvent of water for injection and dimethyl sulfoxide (DMSO; 0.03%), and a specific volume of PTX solution (150 μL) was systemically injected. The hydrogel-based samples were locally injected into the mice. The hydrogel precursor solutions were added to a 1-mL insulin syringe, photopolymerizable using blue light irradiation for 10 s, and then injected near the tumor-bearing tissue in mice. The change in cancer volume and body weight of the mice administered with each treatment were measured once every 2 days. The cancer volume was calculated using the following formula: V = 0.5 × longest diameter × (shortest diameter)^2^. Histological evaluation was performed using hematoxylin and eosin (H&E) stains. Tumor and liver tissues from each mouse were extracted and dipped into formaldehyde (4 *v/v*%) for 1 day. After dehydrating the tissue in a series of ethanol solutions, it was embedded in paraffin until blocking occurred and then sliced in 3 μm-thick sections. These sections were observed using a slide scanner (Pannoramic MIDI; 3DHISTECH Ltd., Budapest, Hungary) and a panoramic viewer (Version 1.15.3; Pannoramic MIDI; 3DHISTECH Ltd., Budapest, Hungary) program.

### 2.9. Statistical Analysis

All quantitative data are expressed as the mean ± standard deviation. One-way analysis of variance (ANOVA) using SPSS Inc. (Chicago, IL, USA) was used for statistical analysis. A value of * *p* < 0.05 was considered statistically significant.

## 3. Results

### 3.1. UV Absorption Spectra

**Figure 2:**
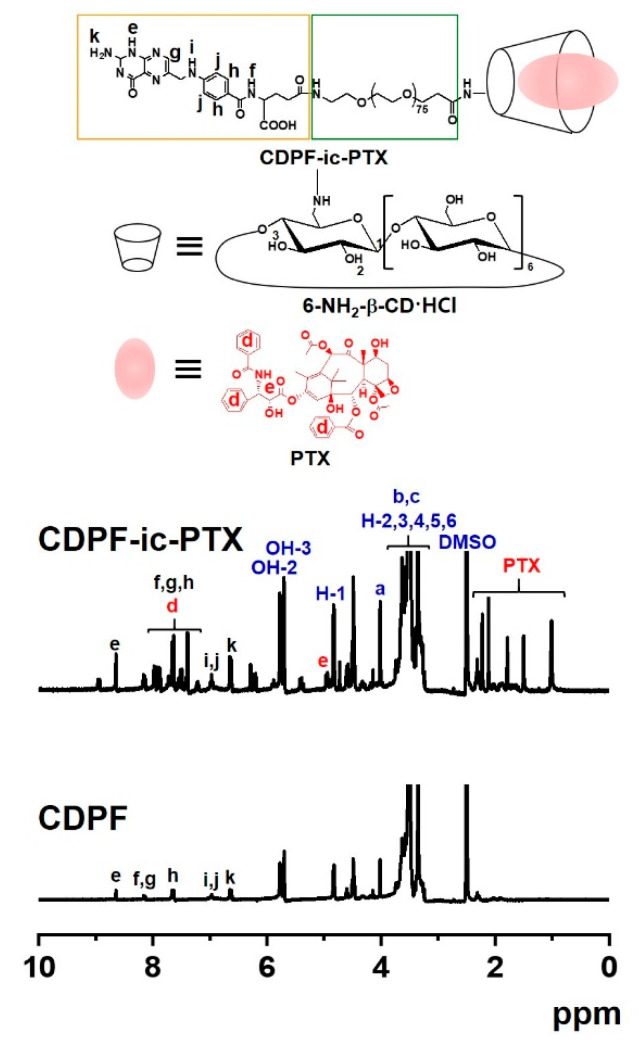
Proton nuclear magnetic resonance (^1^H NMR) spectra of CDPF and CDPF-ic-PTX, which were analyzed using DMSO-*_d6_*.

The ^1^H NMR analysis of CDPF, CDPF-ic-PTX, CDP, and CDP-ic-PTX was performed using DMSO-*_d6_* and D_2_O (Figure 2 and Figure S1). In the ^1^H NMR spectrum of CDPF in DMSO-*_d6_*, the conjugation of 6-NH_2_-β-CD⋅HCl to FA-PEG-COOH formed new peaks at 5.67–5.86 ppm, 4.82 ppm, 4.48 ppm, and 3.19–3.86 ppm assigned for the OH-2,3, H-1, OH-6, and H-2,3,4,5,6 of the ring molecule, and 6.64 ppm, 6.97 ppm, 7.65 ppm, 8.16 ppm, and 8.65 ppm assigned for folic acid. The integration between 4.82 ppm and 7.65 ppm indicated that the conjugation ratio of 6-NH_2_-β-CD⋅HCl was almost 1 to 1 (Figure S1). The ^1^H NMR analysis of PTX in D_2_O is practically impossible due to its poor water solubility. The ^1^H NMR spectrum of CDPF-ic-PTX in D_2_O exhibited the peaks of PTX at 1.00 ppm, 1.15 ppm, and around 8.00 ppm (Figure S1). The inclusion complex between β-CD and PTX makes the drug soluble in water; therefore, the ^1^H NMR analysis of the drug is possible in D_2_O [20]. In addition, in the 1H NMR spectrum of CDPF-ic-PTX in DMSO-*_d6_*, peaks assigned for PTX was observed between 0.89–2.39 ppm and between 7.16–8.23 ppm. In addition, CDP showed the unique peaks of 6-NH_2_-β-CD⋅HCl and PEG at the same positions (Figure S1). From the results of UV-vis spectroscopy, we could expect that PTX was included in the ring molecules of CDPF-ic-PTX and CDP-ic-PTX in molar ratios of 1:1 and 1:2. PTX in CDPF-ic-PTX was complexed in molar ratios of 70% 1:1 and 30% 1:2. PTX in CDP-ic-PTX was complexed in molar ratios of 10% 1:1 and 90% 1:2 (Figure 1 and Figure S1) [21].

### 3.2. UV Absorption Spectra

**Figure 3:**
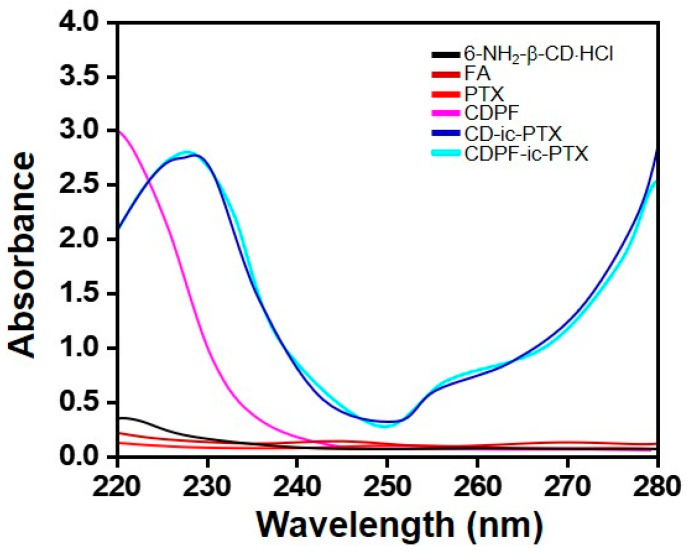
UV spectra of 6-monodeoxy-6-monoamino-β-cyclodextrin⋅hydrochloride (6-NH_2_-β-CD⋅HCl), folic acid (FA), PTX, CDPF, CD-ic-PTX, and CDPF-ic-PTX monitored from 220 nm to 280 nm. The inclusion complex between CDPF and PTX made the specific absorption peak (228 nm) of PTX appear due to the improved water solubility of the drug.

PTX is known to have an absorption peak of 230 nm in solvents that can dissolve the drug, but it did not show its specific absorption peak in PBS due to its poor water solubility [19]. To further investigate the inclusion complexes between CD/CDPF and PTX, UV-vis spectroscopy using PBS (pH 7.4) was employed (Figure 3). Extinct absorption peaks were not observed in the spectra of 6-NH_2_-β-CD⋅HCl, CD, and CDPF from 220 nm to 280 nm. PTX did not show its remarkable absorption peak in PBS because of its poor water solubilities, but in CD-ic-PTX and CDPF-ic-PTX, obvious peaks at 228 nm were observed, indicating that PTX was included into β-CDs of CD and CDPF.

### 3.3. Differential Scanning Calorimetry (DSC) Curves

**Figure 4:**
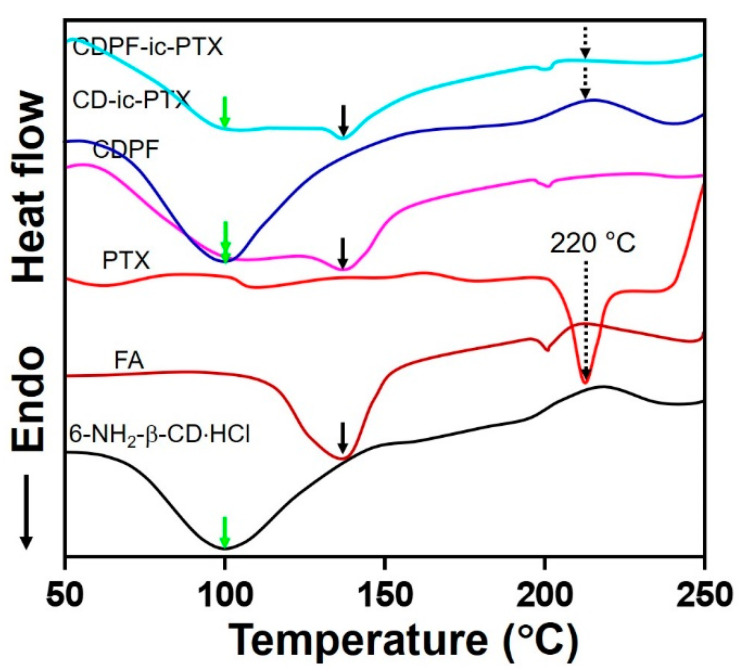
Differential scanning calorimetry (DSC) curves of 6-NH_2_-β-CD⋅HCl, FA, PTX, CDPF, CD-ic-PTX, and CDPF-ic-PTX monitored from 50 °C nm to 400 °C. The inclusion complex between CDPF and PTX made the specific endothermic peak (220 °C) of PTX disappear. The green, black, and black dotted lines indicate the endothermic peak positions of 6-NH_2_-β-CD⋅HCl, FA, and PTX, respectively. Due to the inclusion complex formation between the ring molecules and PTX, CD-ic-PTX, and CDPF-ic-PTX exhibited the disappearance of the endothermic peak of PTX.

The inclusion complex formation of CD/CDPF and PTX was further investigated by DSC analysis, monitoring from 50 °C to 250 °C (Figure 4 and Figure S2). In common with β-CD, 6-NH_2_-β-CD⋅HCl showed a broad endothermic peak around 100 °C. FA has two endothermic peaks at 138 °C and 201 °C [22]. The DSC curves of CDPF, CD-ic-PTX, and CDPF-ic-PTX, showed the endothermic peaks of 6-NH_2_-β-CD⋅HCl and FA. As shown in the DSC curve of PTX, their unique endothermic peaks were observed in 220 °C, respectively [20,22]. However, the endothermic peak of PTX was not observed in CD-ic-PTX and CDPF-ic-PTX. Pak et al. reported the DSC curve of PTX-complexed dimethyl-β-CD (DM-β-CD) [23]. Considering this previous report, the disappearance of the endothermic peak of PTX in CD-ic-PTX and CDPF-ic-PTX may be attributed to the inclusion complex formation between DM-β-CD and PTX [20,23].

### 3.4. Swelling Ratio

The swelling ratios of MGC/PTX, MGC/CD-ic-PTX, and MGC/CDPF-ic-PTX hydrogels, measured at 37 °C for 7 days in PBS (pH 7.4), are shown in Figure S3. All hydrogels displayed similar swelling ratios for the periods tested. The hydrogels exhibited a gradual increase in the swelling ratio for 3 days because of the penetration of water molecules. After that, swelling of the hydrogels was not observed. The swelling ratio is dependent on the degree of cross-linking density [24]. Therefore, it is reasonable to say that hydrogels with the same degree of cross-linking density have the same swelling behavior.

### 3.5. Release Behavior

**Figure 5:**
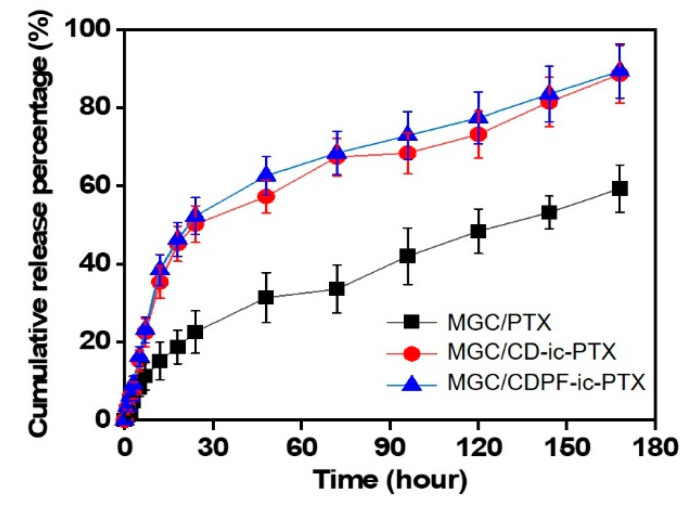
Release behavior profile of PTX in MGC/PTX, MGC/CD-ic-PTX, and MGC/CDPF-ic-PTX photocured by visible light irradiation for 10 s in PBS (pH 7.4), which was measured for 7 days. Three experiments were performed (*n* = 3).

Our previous study proved that the visible light-cured MGC hydrogel not only made it easy to load the anti-cancer drugs in the hydrogel precursor solution before photocuring but also showed a sustained release behavior by drug diffusion from the bulk to the surface of the hydrogel, resulting from the structural properties of the hydrogel after photocuring [2,3,4,5]. The release behavior of PTX in MGC/PTX, MGC/CD-ic-PTX, and MGC/CDPF-ic-PTX at 37 °C for 3 days using PBS (pH 7.4) was investigated (Figure 5). All hydrogels exhibited a similar release behavior, with both an initial burst and sustained release. For MGC/PTX, PTX was rapidly released within 12 h and was sustainably released for 7 days. For MGC/CD-ic-PTX and MGC/CDPF-ic-PTX, the initial burst was observed within 48 h, and sustained release was observed thereafter. These results may be ascribed to the effect of MGC, β-CD, and CDPF, as the drug reservoirs enhance the water solubility of PTX, leading to the rapid release of PTX. In addition, the MGC could induce drug diffusion from the matrix. The 3-D network is swollen by the penetration of water molecules, and the drugs are released to the outside by diffusion through the swollen structure [25]. Thus, the drugs close to the hydrogel surface show a rapid release behavior, and the drugs in the bulk phase are released in a sustained manner by a concentration gradient.

### 3.6. In Vitro Anti-Cancer Effect

**Figure 6:**
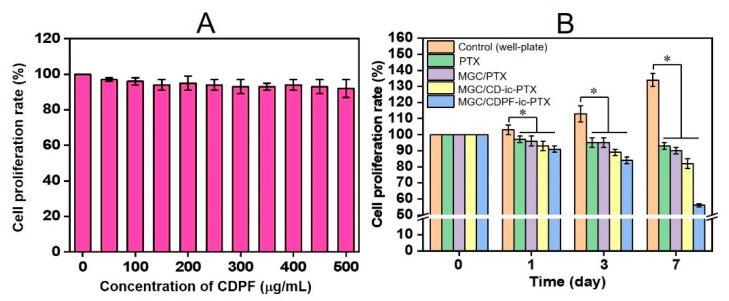
In vitro viability of MCF-7 cells cultured on (**A**) various concentrations of CDPF, and (**B**) MGC/PTX, MGC/CD-ic-PTX, and MGC/CDPF-ic-PTX photopolymerizable by visible light irradiation for 10 s, as compared with that of control (untreated). The cell proliferation rates of PTX, MGC/PTX, MGC/CD-ic-PTX, and MGC/CDPF-ic-PTX were compared to that of control at days 1, 3, and 7 (* *p* < 0.05). The cell viability rate (%) was determined by CCK-8 assay at 1, 3, and 7 days. Three experiments were performed (*n* = 3).

In vitro cell proliferation tests of MCF-7 cells cultured with various concentrations of CDPF, and PTX, MGC/PTX, MGC/CD-ic-PTX, and MGC/CDPF-ic-PTX were conducted to evaluate their anti-cancer activity in comparison with that of control (Figure 6). As shown in Figure 6A, CDPF ranging from 0 to 500 μg/mL had no cytotoxicity, indicating its biocompatibility. The control exhibited a gradual increase in the cell proliferation rate for 7 days, but PTX-based samples gradually decreased over the test period (Figure 6B). In addition, MGC/CDPF-ic-PTX had a superior anti-cancer effect among the PTX-based samples. The cell proliferation rates of PTX-, MGC/PTX-, MGC/CD-ic-PTX-, and MGC/CDPF-ic-PTX-treated samples at 7 days decreased 7, 9, 17, and 42% of those at day 0. These results may be affected by the cellular uptake difference between CD-ic-PTX and CDPF-ic-PTX; therefore, flow cytometry analysis was carried out (Figure S4). Because of the no fluoresce of PTX, Cou6 was used. We found that the cellular uptake of CDPF-ic-Cou6 on MCF-7 cells was higher than that on NIH3T3-E1 cells. In addition, it was found that FA and PEG had a significant role in improving the cellular uptake on MCF-7 cells. These results indicated that CDPF is a good carrier for effectively delivering PTX into MCF-7 cells, followed by the lowest cell proliferation rate.

### 3.7. In Vivo Anti-cancer Effect

Although chemotherapy has been widely used as a main method for cancer therapy, it often has limitations on the improvement in the success rate due to the low targetability of cancer drugs to tumor tissues. Clinically used drug administration methods have many barriers to reaching cancer tissues. For example, in the case of intravenous injection, the anti-cancer drugs injected through the blood vessels first encounter plasma proteins, which may cause the loss of the drugs to some degree [26,27,28,29]. In addition, the drugs can reach normal cells, causing various side effects. In this respect, delivering a large number of drugs to cancer tissues with minimum loss may be one of the most fundamental factors in cancer treatment. The injectable MGC hydrogel proposed in our previous studies facilitated the accessibility of anti-cancer drugs to cancer tissues by enabling injection around cancer tissues.

Another consideration for cancer treatment is that the anti-cancer drugs delivered in cancer tissues must be internalized into the cancer cells to induce cell apoptosis, followed by the promotion of cancer treatment. In chemotherapy, the targeted cancer treatment is mostly dependent on active targeting [30], which relies on the capability of the targeting agents or ligands possessing a strong affinity with cancer cells [31,32]. This specific ligand–receptor binding will lead to the delivery of anti-cancer drugs to tumor tissue. Here, we demonstrated a drug delivery system consisting of MGC hydrogel photopolymerizable by visible light and CDPF-based nanocarrier for PTX delivery, in which the MGC and CDPF were found to be good platforms for targeted drug delivery and targeted cancer therapy, respectively [2,3,4,5,10,11].

Our previous study reported the feasibility of CDPF as a ligand-conjugated PTX carrier for improving active targeting with cancer cells [11]. Among the components of CDPF, folic acid is a vitamin compound (B9) with a low molecular weight that has effective tumor targeting, such as breast, lung, kidney, ovarian, and epithelial mouth, with overexpressed folate receptor-related cancers [33]. PEGylation is known to affect the cellular uptake of nanoparticles into cancer cells [34]. Cruje et al. reported that the short chain lengths of PEG lead to high cellular uptake [34]. In this study, 6-NH_2_-β-CD⋅HCl was conjugated to a compound consisting of PEG and folic acid. In this study, flow cytometry analysis proved that folic acid and PEG have a significant influence on improving the cellular uptake in MCF-7 cells (Figure S3). β-CD has a hydrophilic ring structure due to its 21 hydroxyl groups and a hydrophobic cavity [35]. Because of its unique cavity structure, many hydrophobic drug molecules of similar size can be included in the hole, leading to the improved water solubility of the drugs [35].

**Figure 7:**
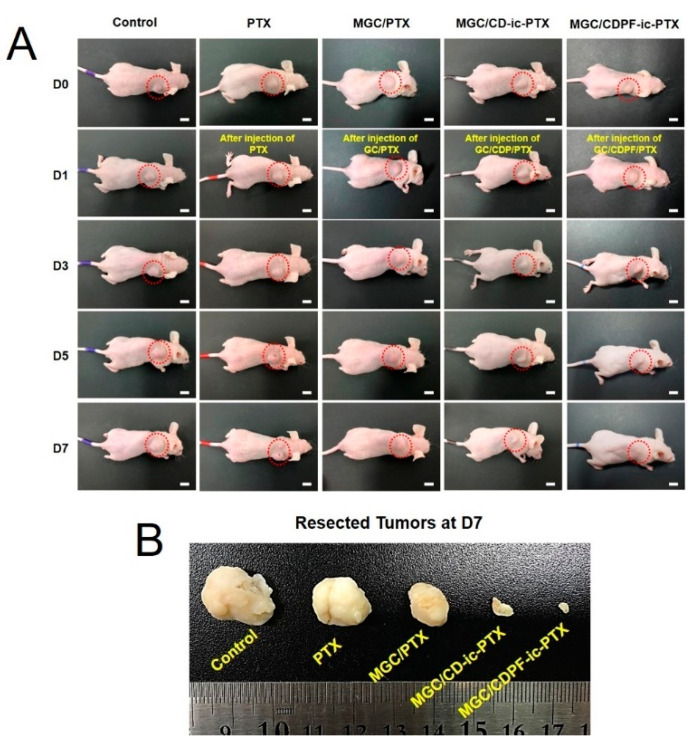
(**A**) Gross appearance of tumor in control, PTX, MGC/PTX, MGC/CD-ic-PTX, and MGC/CDPF-ic-PTX treated mice on day 0, 1, 3, 5, and 7. (**B**) Comparison of tumor size extracted from control, PTX, MGC/PTX, MGC/CD-ic-PTX, and MGC/CDPF-ic-PTX-treated mice at day 7.

The in vivo anti-cancer activity of MGC/CDPF-ic-PTX against breast cancer using the cancer-bearing xenograft mouse model was compared with those of PTX, MGC/PTX, and MGC/CD-ic-PTX (Figure 7). As shown in Figure 7A, the gross appearances of cancers in the mice were observed for 0, 1, 3, 5, and 7 days. The MGC-based samples exhibited a remarkable tumor volume decrease with an increase in time. Conversely, the tumor volumes of control and PTX samples increased gradually. The tumor tissues of all groups at day 7 are shown in Figure 7B. The tumor volumes in MGC/PTX-, MGC/CD-ic-PTX-, and MGC/CDPF-ic-PTX-treated mice were smaller than those in the control and PTX-treated mice. An obvious decrease in the tumor volume was found in MGC/CD-ic-PTX- and MGC/CDPF-ic-PTX-treated mice. Furthermore, MGC/CDPF-ic-PTX resulted in the smallest tumor volume, which can be ascribed to the improvement in the specific targeting of PTX against cancer cells through receptor-mediated endocytosis of CDPF. These findings indicate the effect of MGC and CDPF on the targeted drug delivery of PTX to breast cancer tissue.

### 3.8. Tumor Volumes and Body Weights

**Figure 8:**
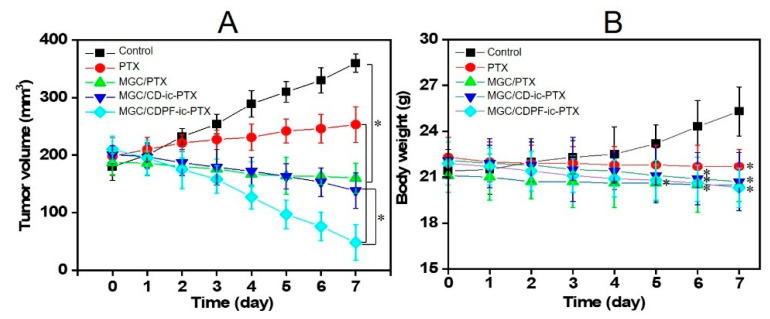
(**A**) Tumor volumes (mm^3^) and (**B**) body weights of control, PTX, MGC/PTX, MGC/CD-ic-PTX, and MGC/CDPF-ic-PTX-treated mice for 7 days. Each group included 3 mice (n = 3; * *p* < 0.05). In the results of tumor volumes, PTX, MGC/PTX, MGC/CD-ic-PTX, and MGC/CDPF-ic-PTX had statistical significance, as compared to control. In addition, a statistical significance was found between MGC/CD-ic-PTX and MGC/CDPF-ic-PTX. In the results of body weights, PTX, MGC/PTX, MGC/CD-ic-PTX, and MGC/CDPF-ic-PTX had statistical significance at day 7, as compared to control.

The tumor volumes of the control and the PTX-based sample-treated mice were calculated (Figure 8A). A gradual increase in the tumor volume was observed in the control and PTX-treated mice. On the contrary, MGC/PTX, MGC/CD-ic-PTX, and MGC/CDPF-ic-PTX resulted in a decrease in tumor volume. Moreover, MGC/CDPF-ic-PTX remarkably decreased the tumor volume. At day 0, the average tumor volumes in the control, PTX-, MGC/PTX-, MGC/CD-ic-PTX-, and MGC/CDPF-ic-PTX-treated mice were 180, 198, 187, 201, and 210 mm^3^, respectively. At day 7, the tumor volumes were 360, 253, 160, 138, and 48 mm^3^, respectively. This result demonstrated the anti-cancer effect of MGC/CDPF-ic-PTX against breast cancer.

Figure 8B shows the body weights of untreated mice and PTX-, MGC/PTX, MGC/CD-ic-PTX, and MGC/CDPF-ic-PTX-treated mice, which were measured for 7 days once a day. In the control, the body weights increased gradually for 7 days, which is probably explained by the rise of the tumor volume. PTX, MGC/PTX, MGC/CD-ic-PTX, and MGC/CDPF-ic-PTX samples led to a gradual decrease for 7 days. Furthermore, MGC/CD-ic-PTX and MGC/CDPF-ic-PTX-treated mice exhibited lower body weights than MGC/PTX-treated mice. In addition, MGC/CDPF-ic-PTX-treated mice exhibited the lowest body weight among all samples tested, which may be attributed to the improved water solubility and cancer cell binding of PTX using CDPF.

### 3.9. Histological Evaluations

**Figure 9:**
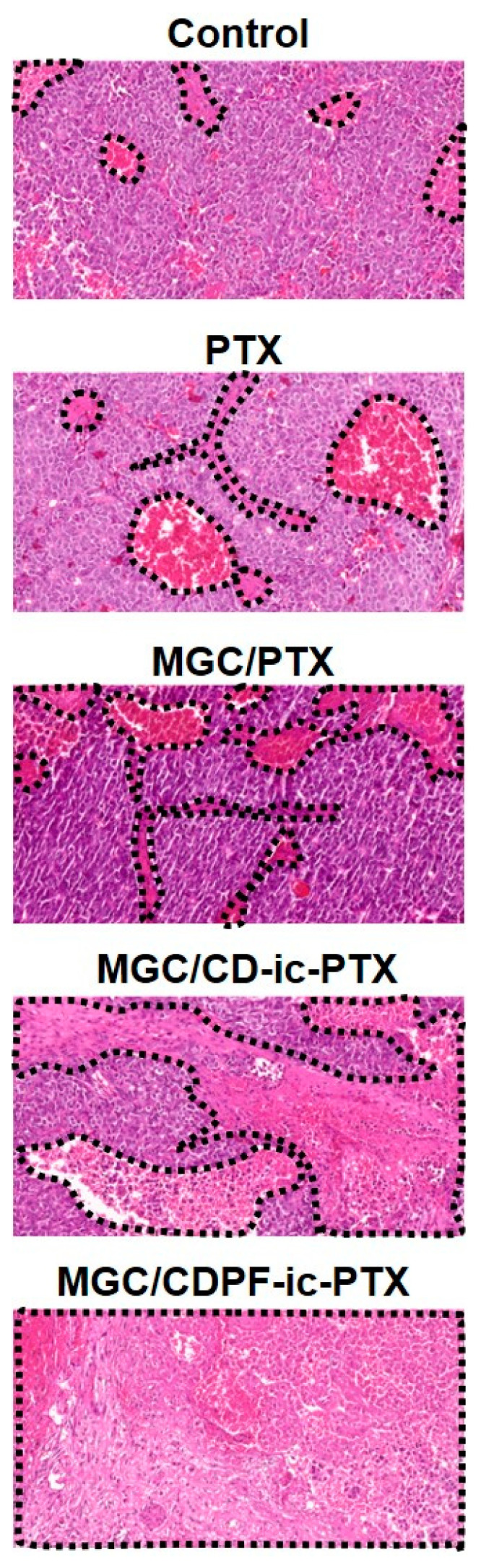
H&E stained images of tumor tissues dissected in control, PTX, MGC/PTX-, MGC/CD-ic-PTX-, and MGC/CDPF-ic-PTX treated mice at 7 days. Black dotted lines indicate necrotic area.

**Figure 10:**
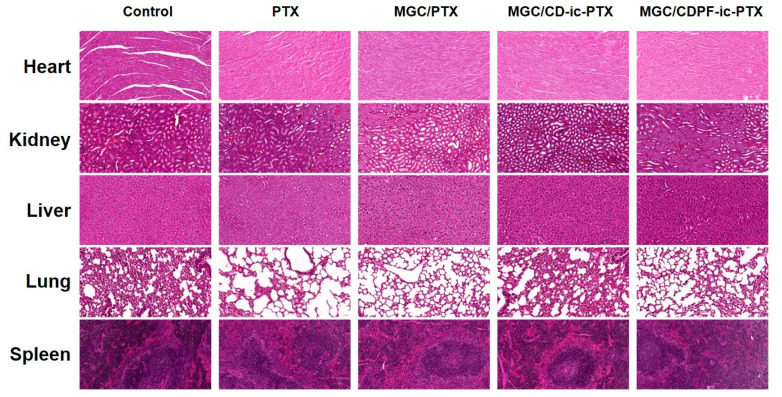
H&E stained images of heart, kidney, liver, lung, and spleen organs dissected in control, PTX, MGC/PTX-, MGC/CD-ic-PTX-, and MGC/CDPF-ic-PTX-treated mice at 7 days.

The H&E staining of cancer tissues treated with PTX-, MGC/PTX-, MGC/CD-ic-PTX-, and MGC/CDPF-ic-PTX was performed and is shown in Figure 9, as compared with that of the control cancer tissue. In no treated cancer tissue (control), densely packed cancer cells were observed overall; conversely, the PTX-based samples induced partial necrosis. Among the samples, MGC/PTX-, MGC/CD-ic-PTX-, and MGC/CDPF-ic-PTX had a greater anti-cancer effect than did PTX. In addition, MGC/CDPF-ic-PTX resulted in a noticeable anti-cancer effect. Figure 10 shows H&E stained images of heart, kidney, liver, lung, and spleen treated with MGC/PTX-, MGC/CD-ic-PTX-, and MGC/CDPF-ic-PTX for 7 days as well as control. The images exhibited no irregular cell/tissue changes. These results suggest the efficacies of the local injection of GC and the improved water-solubility and cancer cell targeting of PTX using CDPF along with cell/tissue compatibility. Our previous study reported the feasibility of injectable visible light-cured GC hydrogels for local and targeted drug delivery systems against cancer treatment [2,3,4,5]. In addition to the feasibility of the GC hydrogel, CDPF-based adamantane/near-infrared fluorophore (ADM-NIRF) was gradually accumulated for 2 days in cancer tissue and demonstrated a gradual decrease for up to 9 days [10]. Conversely, ADM-NIRF disappeared within 2 days [10]. These results indicate that folic acid acts as an important factor for binding with breast cancer cells. As expected, through histological evaluations, we showed that injectable visible light-cured GC hydrogel/CDPF-based drug carriers increase the accumulation of PTX in cancer tissues and the uptake of the drug into cancer cells, leading to improved anti-cancer effects. This combined system may contribute to the high delivery rate of PTX into cancer tissue and the improved cellular uptake of PTX in cancer cells.

## 4. Conclusions

This study reported that the anti-cancer effect of MGC/CDPF-ic-PTX against breast cancer in vivo, as compared to those of control (no treatment), PTX, MGC/PTX, and MGC/CD-ic-PTX. Preparation of MGC/CD-ic-PTX was characterized by ^1^H NMR, UV-vis, and DSC analyses. MGC/PTX, MGC/CD-ic-PTX, and MGC/CDPF-ic-PTX exhibited swollen states for 3 days and equilibrium states thereafter, and the controlled release behaviors of PTX for 7 days. Results of cell viability and animal study revealed that MGC/CDPF-ic-PTX had a larger anti-cancer effect than PTX, MGC/PTX, and MGC/CD-ic-PTX because PTX can be locally delivered to tumor tissue by the injectable MGC hydrogel and can be specifically internalized into breast cancer cells by receptor-mediated endocytosis.

## Supplementary Materials

The following are available online at https://www.mdpi.com/2079-4991/11/2/317/s1, Figure S1: ^1^H NMR spectrum; Figure S2. DSC curves of 6-NH_2_-β-CD⋅HCl, FA, PTX, CDPF, CD-ic-PTX, and CDPF-ic-PTX monitored from 50 °C nm to 400 °C; Figure S3. Swelling ratio of MGC/PTX, MGC/CD-ic-PTX and MGC/CD-ic-PTX photopolymerizable by visible light irradiation for 10 s, which was measured for 7 days. Figure S4. Flow cytometry assay of control (MCF-7), CD-ic-PTX/CDP-ic-PTX/CDPF-ic-PTX-treated MCF-7, and CDPF-ic-PTX-treated NIH3T3-E1.
