# Supplementary material for: Injectable Glycol Chitosan Hydrogel Containing Folic Acid-Functionalized Cyclodextrin-Paclitaxel Complex for Breast Cancer Therapy
Source: Nanomaterials (Basel). 2021 Jan 27;11(2):317. doi: 10.3390/nano11020317 (PMC7910883; doi:10.3390/nano11020317)
Supplement: Supplementary file 1 [file nanomaterials-11-00317-s001.pdf]

nanomaterials

Supplementary Materials

Injectable Glycol Chitosan Hydrogel Containing Folic Acid-Functionalized Cyclodextrin-Paclitaxel Complex for Breast Cancer Therapy

Hoon Hyun [1,†], Min Ho Park [2,†], Gayoung Jo [1], Bo Young Lee [1], Jae Won Choi [3], Heung Jae Chun [4,5], Hyeon Soo Kim [5] and Dae Hyeok Yang [5,*]

[1] Department of Biomedical Sciences, Chonnam National University Medical School, Gwangju 61469, Korea; hhyun@jnu.ac.kr (H.H.); 176250@jnu.ac.kr (G.J.); 196729@jnu.ac.kr (B.Y.L.)
[2] Department of Surgery, Chonnam National University Medical School, Gwangju 61469, Korea; mhpark@jnu.ac.kr
[3] Lumenbio Co. LTD., Seoul 08590, Korea; aa@lumenbio.co.kr
[4] Department of Biomedical & Health Sciences, College of Medicine, The Catholic University of Korea, Seoul 06591, Korea; chunhj@catholic.ac.kr
[5] Institute of Cell and Tissue Engineering, College of Medicine, The Catholic University of Korea, Seoul 06591, Korea; chunhj@catholic.ac.kr (H.J.C.); 21905010@cmcnu.or.kr (H.S.K.)
* Correspondence: yangdh@catholic.ac.kr; Tel.: +82-2-2258-7497
† These authors contributed equally to this work.

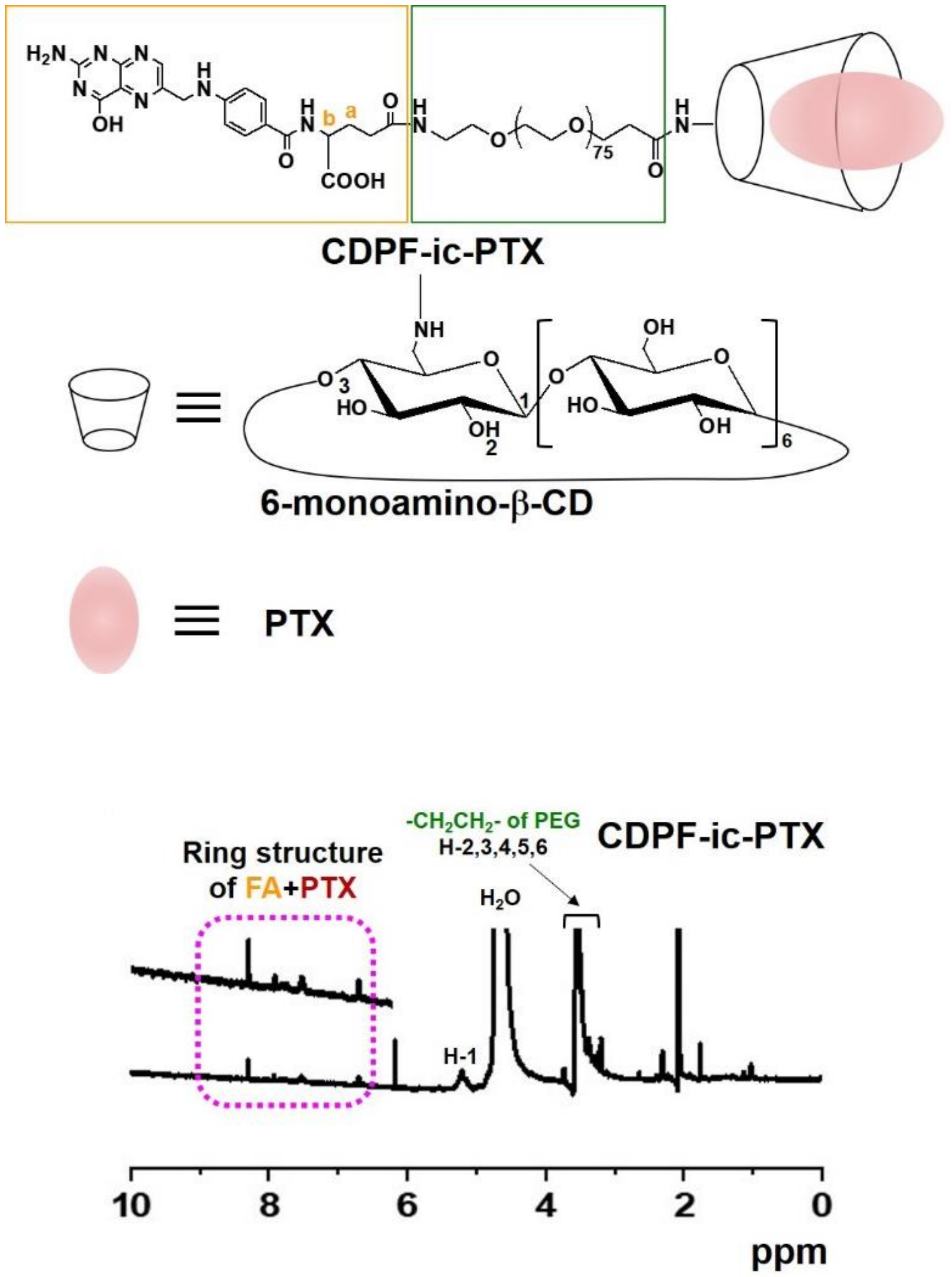
CDPF-ic-PTX
6-monoamino-β-CD
PTX
-CH2CH2- of PEG
H-2,3,4,5,6
CDPF-ic-PTX
Ring structure
of FA+PTX
H2O
H-1
ppm

(a)

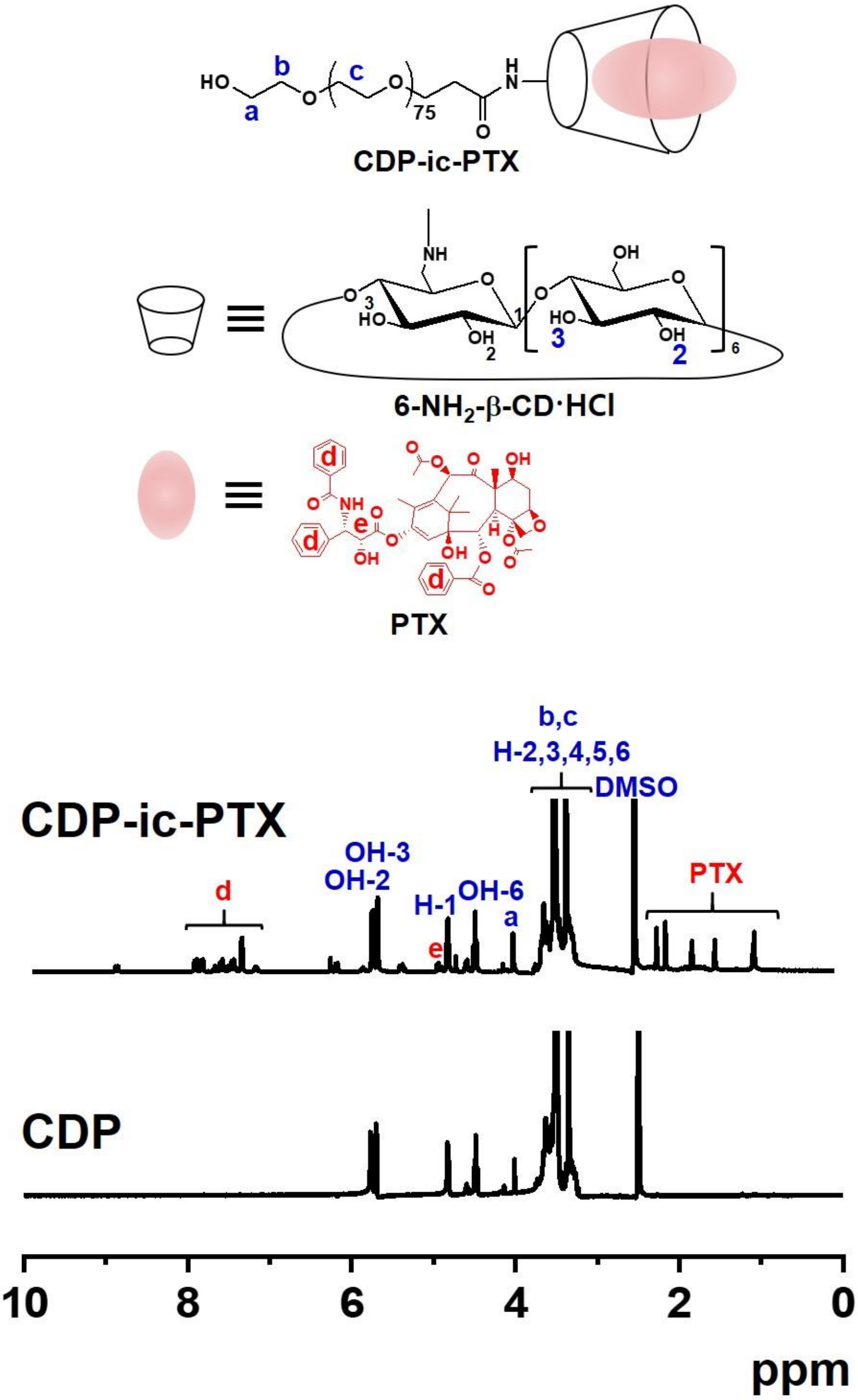
CDP-ic-PTX
6-NH2-β-CD·HCl
PTX
CDP-ic-PTX
CDP
b,c
H-2,3,4,5,6
DMSO
OH-3
OH-2
H-1
OH-6
a
d
e
PTX
10
8
6
4
2
0
ppm

(b)

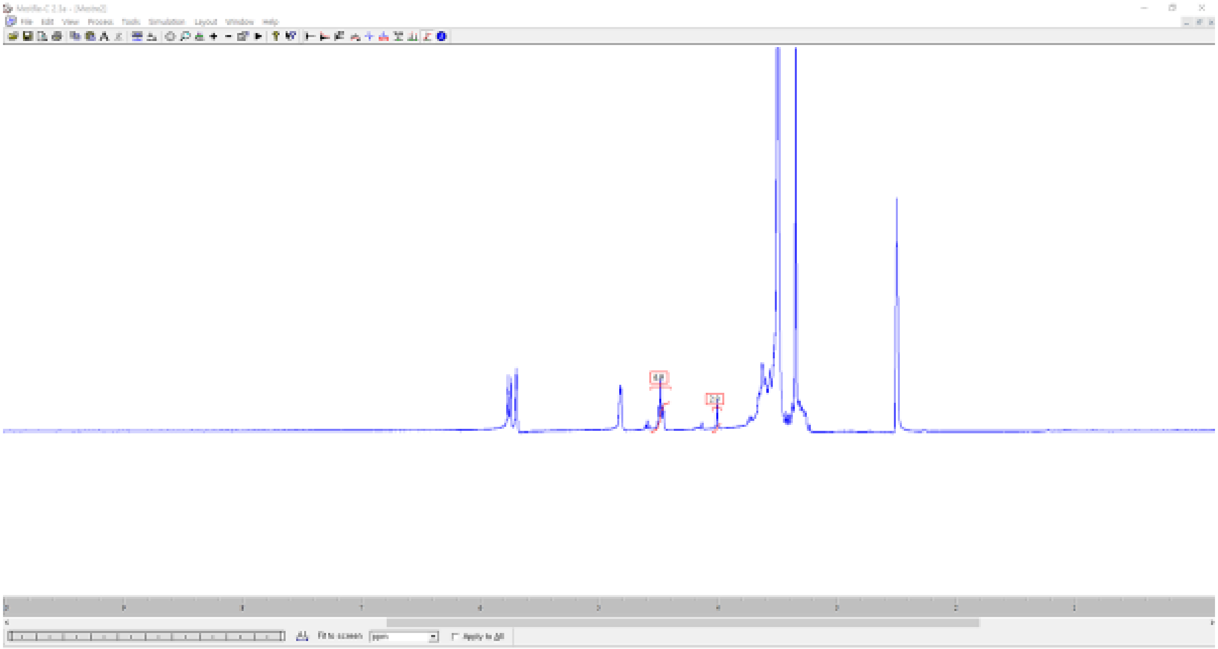

(c)

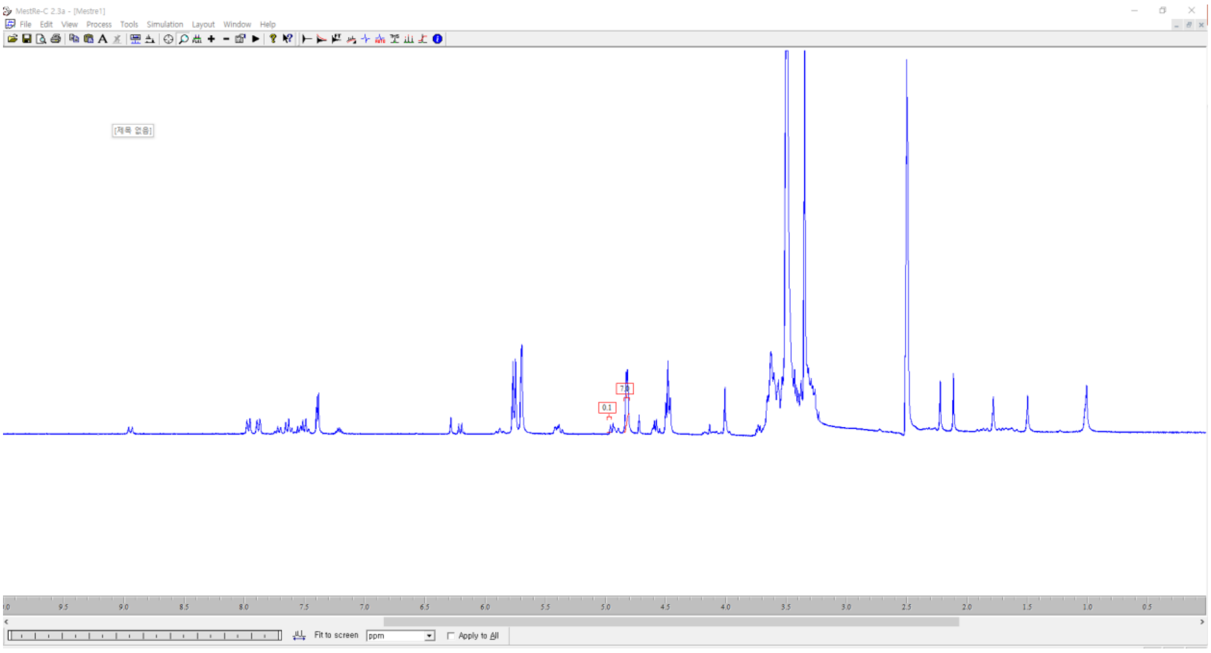
MestRe-C 2.3a - [Mestre1]
File Edit View Process Tools Simulation Layout Window Help
[제목 없음]
7.0
0.1
9.5
9.0
8.5
8.0
7.5
7.0
6.5
6.0
5.5
5.0
4.5
4.0
3.5
3.0
2.5
2.0
1.5
1.0
0.5
Fit to screen
ppm
Apply to All

(d)

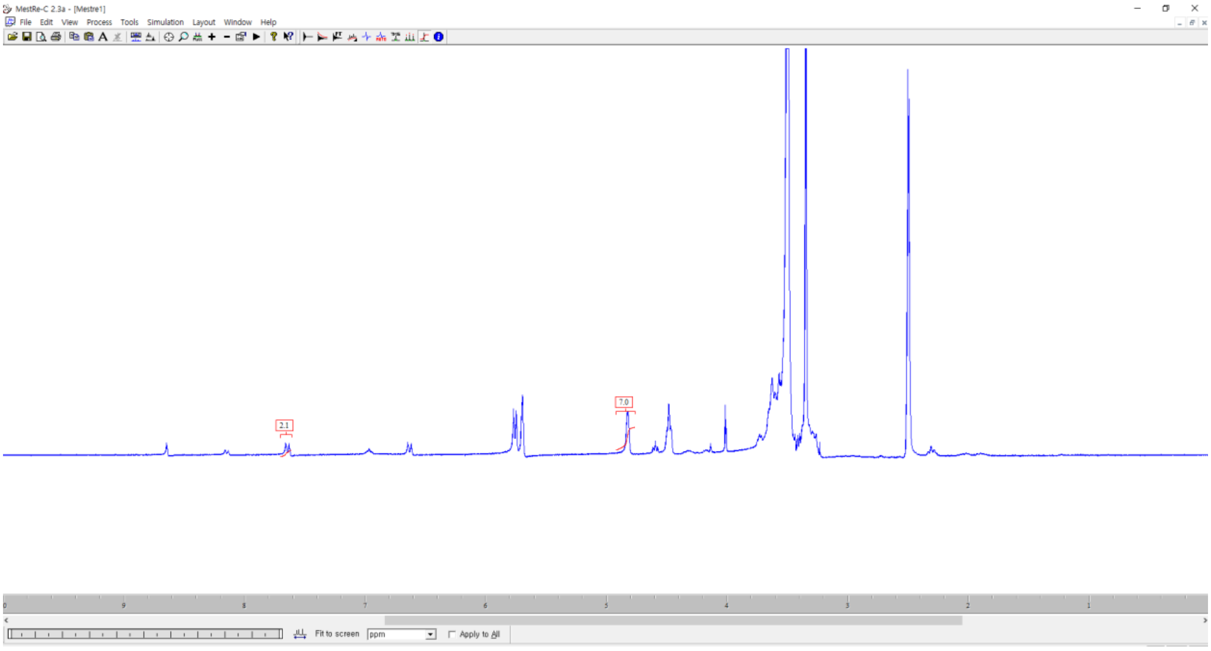
MestRe-C 2.3a - [Mestre1]
File Edit View Process Tools Simulation Layout Window Help
2.1
7.0
9
8
7
6
5
4
3
2
1
Fit to screen
ppm
Apply to All

(e)

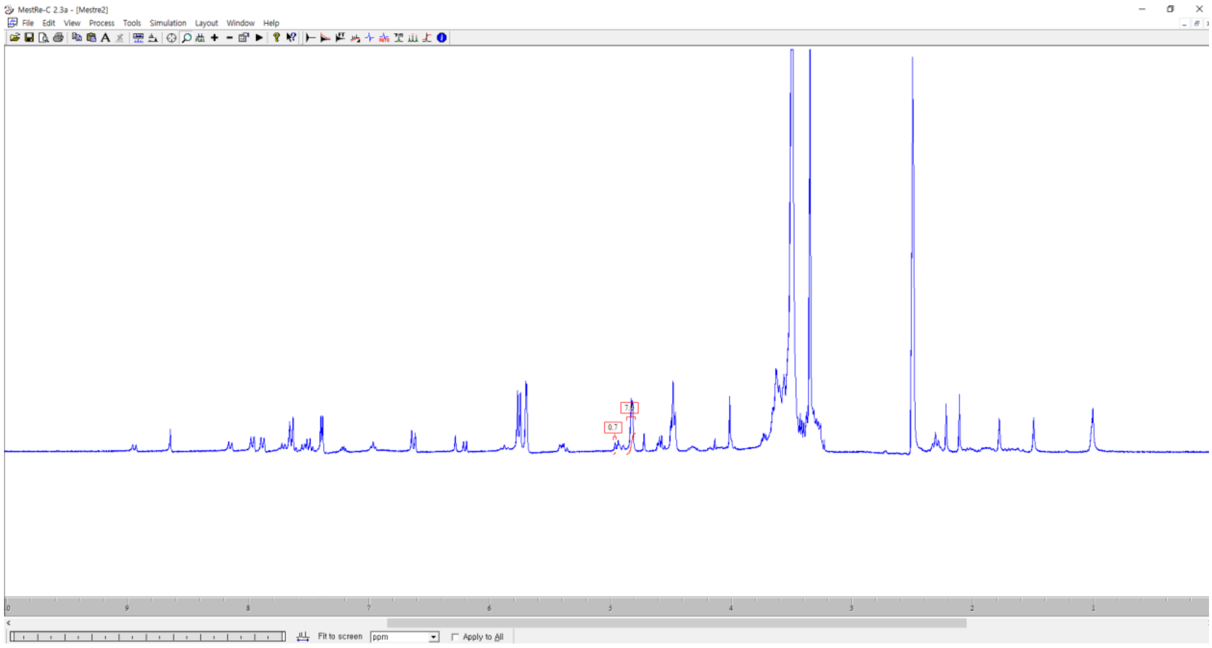

(f)

Figure S1. (**a**) ^{1}H NMR spectrum of CDPF-ic-PTX analyzed using D_2O. (**b**) ^{1}H NMR spectra of CDP and CDP-ic-PTX analyzed using DMSO-d_6. (**c**) ^{1}H NMR spectrum of CDP. (**d**) ^{1}H NMR spectrum of CDP-ic-PTX. (**e**) ^{1}H NMR spectrum of CDPF. (**f**) ^{1}H NMR spectrum of CDPF-ic-PTX.

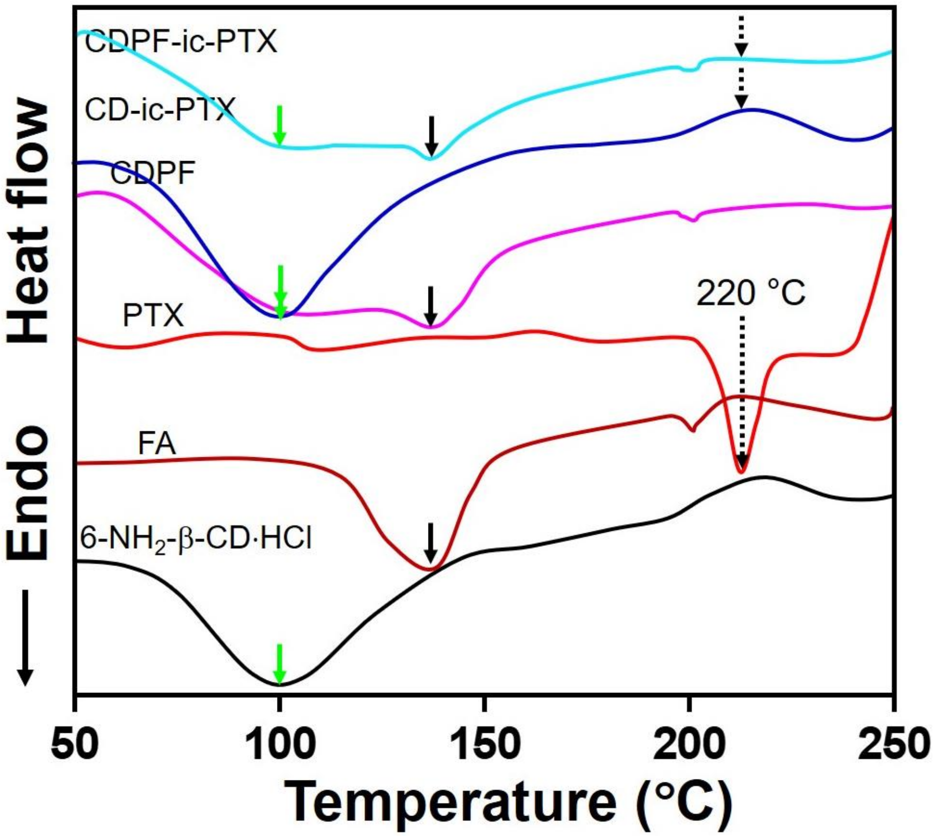

Figure S2. DSC curves of 6-NH2-β-CD·HCl, FA, PTX, CDPF, CD-ic-PTX, and CDPF-ic-PTX monitored from 50 °C nm to 400 °C. Inclusion complex between CDPF and PTX made the specific endothermic peak (220 °C) of PTX disappear. The green, black, and black dotted lines indicate the endothermic peak positions of 6-NH2-β-CD·HCl, FA, and PTX, respectively. Due to the inclusion complex formation between the ring molecules and PTX, CD-ic-PTX, and CDPF-ic-PTX exhibited the disappearance of endothermic peak of PTX.

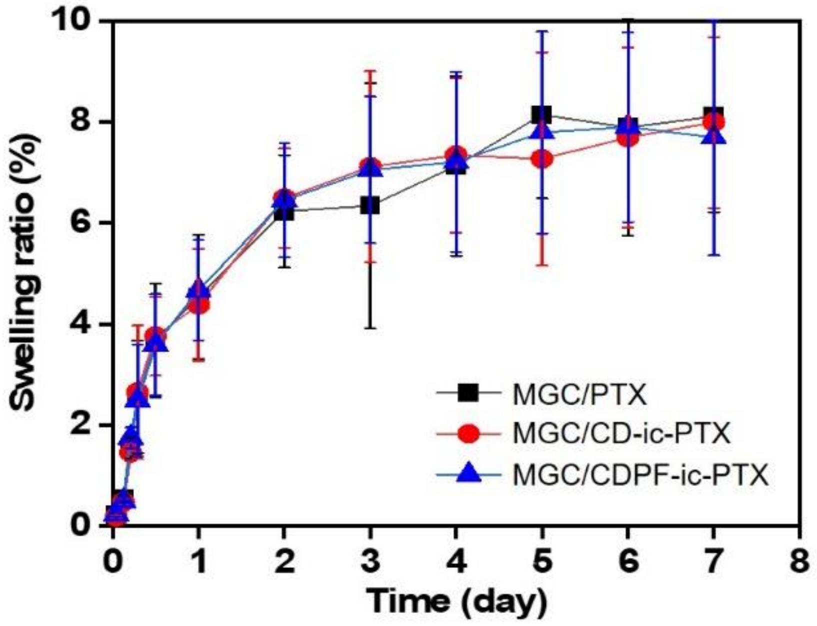

Figure S3. Swelling ratio of MGC/PTX, MGC/CD-ic-PTX and MGC/CD-ic-PTX photopolymerizable by visible light irradiation for 10 s, which was measured for 7 days. Three experiments were performed (n = 3).

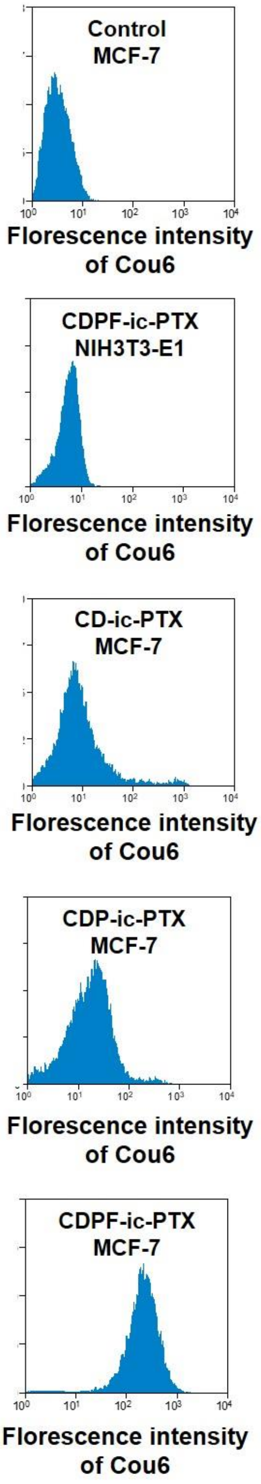

Figure S4. Flow cytometry assay of control (MCF-7), CD-ic-PTX/CDP-ic-PTX/CDPF-ic-PTX-treated MCF-7, and CDPF-ic-PTX-treated NIH3T3-E1.

© 2021 by the authors. Licensee MDPI, Basel, Switzerland. This article is an open access article distributed under the terms and conditions of the Creative Commons Attribution (CC BY) license (http://creativecommons.org/licenses/by/4.0/).
